# Post-procedural myocardial infarction following surgical and trans-catheter aortic valve replacement - mechanistic insights from cardiovascular magnetic resonance imaging

**DOI:** 10.1186/1532-429X-18-S1-P337

**Published:** 2016-01-27

**Authors:** Laura E Dobson, Tarique A Musa, Akhlaque Uddin, Timothy A Fairbairn, Peter Swoboda, David P Ripley, Adam K McDiarmid, Bara Erhayiem, Pankaj Garg, Betsy Evans, Christopher Malkin, Daniel Blackman, Sven Plein, John P Greenwood

**Affiliations:** 1 Cardiac MRI, Leeds Institute for Cardiovascular and Metabolic Medicine, Leeds, United Kingdom; 2 grid.415967.80000000099651030Leeds Teaching Hospitals NHS Trust, Leeds, United Kingdom

## Background

Cardiac biomarker release is ubiquitous following surgical and transcatheter aortic valve replacement (SAVR and TAVR), preventing accurate discrimination between release due to focal myocardial infarction (MI) and global myocardial injury. Cardiovascular magnetic resonance (CMR) late gadolinium enhancement imaging (LGE) is the most sensitive imaging method to detect post-procedural new MI. Our study aimed to compare rates of new MI using CMR LGE before and 6m after TAVR and SAVR.

## Methods

Ninety six patients with severe aortic stenosis undergoing TAVR (n = 57) and SAVR (n = 39) were prospectively recruited and scanned prior to (median 1 day) and 6 months following valve intervention. The presence of significant coronary artery disease (CAD) was determined by the occurrence of a >50% stenosis in any major epicardial vessel. Areas of LGE were quantified with computer-assisted planimetry (2SD; cmr^42^ ,Circle CVI). Presence of new LGE was determined by direct comparison of pre and post-procedure scans.

## Results

The SAVR group was younger, less symptomatic, had less 3 vessel coronary artery disease (CAD) and were at lower surgical risk than the TAVR group. Most (87%) SAVR implants were bioprosthetic and most TAVR implants were Medtronic CoreValve (79%) (86% Transfemoral). Thirty-four (60%) patients had non-revascularized CAD at the time of TAVR. MI pattern LGE was present at baseline in 24 TAVR (42%) and 9 SAVR (24%) patients.The rate of new MI was greater in the SAVR group than the TAVR group (SAVR, n = 10(26%) vs. TAVR, n = 3(5%), p = 0.004). Absolute mean infarct mass was similar between groups (SAVR 1.1 g ± 0.6 g vs. TAVR 2.0 g ± 1.4 g, p = 0.395) as was infarct mass as a percentage of left ventricular mass (SAVR 1.0 ± 0.4% vs. TAVR 2.2 ± 1.3%, p = 0.268) (Figure 2). None of the SAVR and only one of the TAVR infarcts were detected clinically. 34 patients (60%) in the TAVR group had non-revascularized coronary artery disease (CAD) at the time of TAVR, of whom only 3 (9%) had new MI. In the SAVR group, 16 patients (41%) underwent concurrent coronary artery bypass grafting (CABG). Patients undergoing CABG were less likely to have a new MI than those not requiring concurrent reavscularisation (CABG 6.3% vs. no CABG 39.1%, p = 0.021) There was no difference in mean cardiopulmonary bypass time (LGE(+) 88.5 ± 31.1 vs. LGE(-) 114.5 ± 47.4 min, p = 0.112) and aortic cross clamp time according to LGE status (LGE(+) 66 ± 25 vs. LGE(-) 84 ± 42 min, p = 0.164).

**Figure 1:**
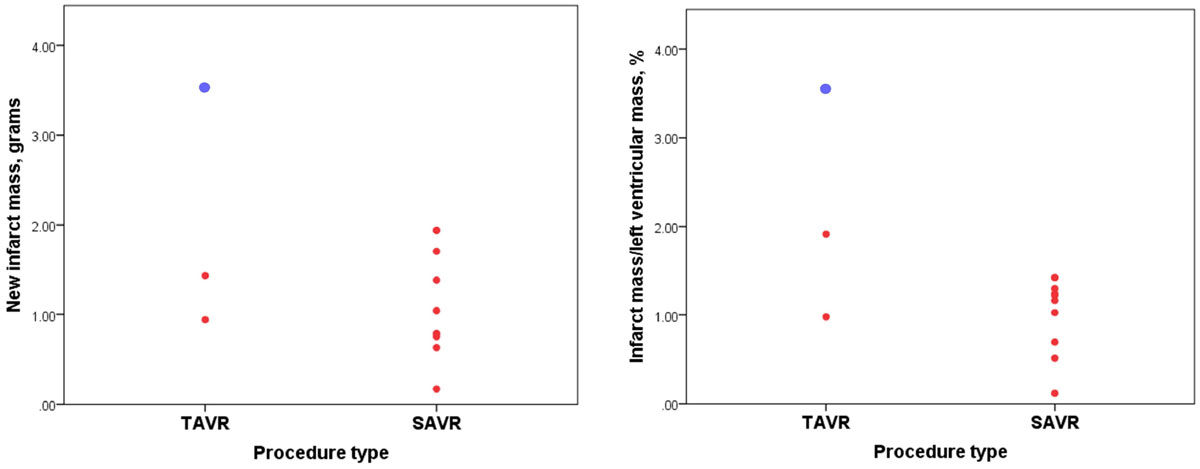
**New infarct mass expressed in absolute terms and as a percentage of left ventricular mass according to procedure type**. The red dots represent individual patients and the blue dot represents the only clinically detected MI according to VARC criteria.

## Conclusions

MI is an infrequent complication of TAVR but is more common following SAVR. Infarct size is small following both procedures. The mechanism is likely to be embolization of valve debris and/or clot. The low new infarct rate in TAVR, especially in the context of high rates of non-revascularized CAD, is reassuring and strengthens the notion that coronary revascularization prior to TAVR may be unnecessary.

